# Suppression of skeletal muscle signal using a crusher coil: A human cardiac ^31^p‐MR spectroscopy study at 7 tesla

**DOI:** 10.1002/mrm.25755

**Published:** 2015-04-28

**Authors:** Benoit Schaller, William T. Clarke, Stefan Neubauer, Matthew D. Robson, Christopher T. Rodgers

**Affiliations:** ^1^ University of Oxford Centre for Clinical Magnetic Resonance Research (OCMR), University of Oxford, Level 0, John Radcliffe HospitalOxfordUnited Kingdom

**Keywords:** B_1_‐insensitive train to obliterate signal (BISTRO), crusher coil, surface spoiling coil, meander coil, ^31^P‐MR spectroscopy, cardiac, saturation bands, chemical shift imaging (CSI)

## Abstract

**Purpose:**

The translation of sophisticated phosphorus MR spectroscopy (^31^P‐MRS) protocols to 7 Tesla (T) is particularly challenged by the issue of radiofrequency (RF) heating. Legal limits on RF heating make it hard to reliably suppress signals from skeletal muscle that can contaminate human cardiac ^31^P spectra at 7T. We introduce the first surface‐spoiling crusher coil for human cardiac ^31^P‐MRS at 7T.

**Methods:**

A planar crusher coil design was optimized with simulations and its performance was validated in phantoms. Crusher gradient pulses (100 μs) were then applied during human cardiac ^31^P‐MRS at 7T.

**Results:**

In a phantom, residual signals were 50 ± 10% with BISTRO (B_1_‐insensitive train to obliterate signal), and 34 ± 8% with the crusher coil. In vivo, residual signals in skeletal muscle were 49 ± 4% using BISTRO, and 24 ± 5% using the crusher coil. Meanwhile, in the interventricular septum, spectral quality and metabolite quantification did not differ significantly between BISTRO (phosphocreatine/adenosine triphosphate [PCr/ATP] = 2.1 ± 0.4) and the crusher coil (PCr/ATP = 1.8 ± 0.4). However, the specific absorption rate (SAR) decreased from 96 ± 1% of the limit (BISTRO) to 16 ± 1% (crusher coil).

**Conclusion:**

A crusher coil is an SAR‐efficient alternative for selectively suppressing skeletal muscle during cardiac ^31^P‐MRS at 7T. A crusher coil allows the use of sequence modules that would have been SAR‐prohibitive, without compromising skeletal muscle suppression. Magn Reson Med 75:962–972, 2016. © 2015 The Authors. Magnetic Resonance in Medicine Published by Wiley Periodicals, Inc. on behalf of International Society of Medicine in Resonance.

## INTRODUCTION

Phosphorus MR spectroscopy (^31^P‐MRS) is a powerful, noninvasive tool to investigate cardiac energy metabolism 1, 2. High‐energy phosphate metabolites, such as adenosine triphosphate (ATP), phosphocreatine (PCr), and inorganic phosphate (Pi), can be detected with ^31^P‐MRS, providing direct insight into myocardial energetics. This has proved beneficial for studying ischemic heart disease, heart failure, cardiomyopathies, and the transplanted heart 3, 4, 5. Nevertheless, clinical applications of cardiac ^31^P‐MRS have so far been limited to research studies due to the low temporal and spatial resolutions achievable at field strengths ≤ 3 Tesla (T) 5.

Theory predicts that the spectral signal‐to‐noise ratio (SNR) increases at higher magnetic fields. This suggests that an increased accuracy and precision of metabolite quantification should be achievable at 7T compared with 3T. This has been confirmed, e.g., for ^1^H‐MRS in the brain 6, 7 and for ^31^P‐MRS in the heart 8. In the heart, a 2.8× increase of PCr SNR and a 2.4× decrease in the Cramér‐Rao Lower Bounds (CRLB) was observed at 7T compared with 3T.

The suppression of contaminating signals in MR spectra (arising from, e.g., extracerebral lipids, skeletal muscle, liver, etc.) has been a concern for decades 9, 10. The contamination level depends on many factors, such as voxel selection performance, motion, and the composition of surrounding tissues 11. In cardiac ^31^P‐MRS, signal from overlying skeletal muscle may contaminate “myocardial” ^31^P‐MR spectra. This can lead to inaccurate quantification 2, and must therefore be avoided. To reduce contamination, additional radiofrequency (RF) pulses are typically inserted into the main localized MR spectroscopy pulse sequence, e.g., spatially selective outer volume suppression pulses 12, 13 or spectrally selective inversion recovery suppression pulses 14, 15.

However, the translation of approaches which involve additional RF pulses to ultra‐high field is challenging. This is because RF energy deposition in tissue increases approximately in proportion to the *square* of the field strength B_0_ 16. Legal regulations on RF‐induced heating or specific absorption rate (SAR) 17, therefore, constrain pulse sequence design at 7T, and can make it impossible to completely suppress contaminating signals 8.

An alternative approach to suppress contaminating signals that does not involve additional RF pulses is to use a surface‐spoiling “crusher coil” instead 18, 19, 20. The principle and basic design of crusher coils was introduced in vitro by Crowley and Ackerman in 1985 18 and demonstrated in vivo by Chen and Ackerman in 1990 for a rat liver MRS study 21, 22. The central idea is that when direct current (DC) is applied to a crusher coil, it produces a region of inhomogeneous magnetic field close to the crusher coil, but it does not perturb the magnetic field further away. Applying a short (∼100 µs) DC pulse to the crusher coil between RF excitation and signal acquisition can dephase any potentially contaminating signals from regions close to the surface, without affecting signals from deeper‐lying regions of interest.

Since that first work, Jehenson and Bloch developed a more rigorous approach to optimize the crusher coil geometry 23. Crusher coils have now been used in two applications in humans: to reduce the field of view (FOV) for ^1^H MRI in the torso in the 1990s 19, 24 and again very recently 25. While our study was in progress, Boer et al 20 have also reported the use of a crusher coil for suppression of lipid signals in human brain ^1^H‐MRS.

In this work, we introduce the first crusher coil for human cardiac ^31^P‐MRS and investigate whether it is an effective replacement for RF saturation bands at 7T. We present below an investigation of the relationship between the spoiling parameters (duration, current) and the residual signal with simulations, followed by a quantitative comparison against B_1_‐insensitive train to obliterate signal (BISTRO) saturation bands 12 in phantoms, before applying the crusher coil in healthy volunteers for a cardiac ^31^P‐MRS study at 7T. We finish by demonstrating how a crusher coil enables the use of SAR‐demanding pulse sequence modules by performing a DANTE (delays alternating with nutations for tailored excitations) 26, 27 selective saturation experiment in the heart at 7 Tesla (T).

## METHODS

### Simulations of Spoiling Field and the Ensuing Signal Suppression

The magnetic field **B_spoil_** (spoiler field) generated by the crusher coil in this study was simulated by integrating over finite wire elements in Matlab (Mathworks 2013a, Natick, MA) using the following integrated form of the Biot‐Savart Law for speed:

(1) $$B_{spoil}=\frac{\mu_{0}I_{spoil}}{4\pi }\hat{e}\times R_{i}\frac{2L(R_{i}+R_{f})}{R_{i}R_{f}}\frac{1}{{\left(R_{i}+R_{f}\right)}^{2}-L^{2}}$$

where μ_0_ is the permeability of free space, I_spoil_ is the current, **ê** is the unit vector along the wire element, L is the length of the wire element, **R**_i(f)_ is the vector from the observation point to the initial (final) end of the wire element and R_i(f)_ = |**R**_i(f)_| 28. After RF excitation, a brief pulse of current I_spoil_ is driven through the crusher coil for a time T_spoil_. The z‐component of this crusher field alters the local Larmor frequency and therefore produces intra‐voxel dephasing. The phase $\varphi_{i}$ of spin isochromat *i* may be defined as:

(2) $$\varphi_{i}=\gamma_{31P}\times B_{i,spoil,z}\times T_{spoil}$$

where γ_31P_ is the ^31^P gyromagnetic ratio and B_i,spoil,z_ is the z‐component of the spoiler field at the location of isochromat *i*. The destruction of the phase coherence between spins within each voxel causes signal suppression, which is most noticeable in voxels near the crusher coil where B_i,spoil,z_ varies rapidly with position.

Simulations were performed for spin isochromats at each of 600 × 600 points uniformly distributed over a simulated region of interest (ROI_sim_) of dimensions 20 × 300 × 300 mm^3^ above the crusher coil plane. The x‐dimension of ROI_sim_ could be set to only 20mm to speed up simulations, because the spoiler field $B_{\mathrm{i,spoil,z}}$varied negligibly along the wire. To quantify the degree of intravoxel spin dephasing generated by the crusher coil, the simulated residual signal in a 20 × 20 × 20 mm^3^ voxel V_sim_ was then derived by integrating the complex spin amplitudes from all spin isochromats *i* contained within that voxel V_sim_:

(3) $$S_{r,sim}(V)=\left|\frac{\sum_{i}e^{i\varphi_{i}}}{\sum_{i}1}\right|\times 100\%\;\;\;\;\;\;\;\;\forall i\in V_{sim}$$

This procedure (Eq. [3]) was then applied repeatedly to sample voxels V_sim_ centered at 1 mm intervals throughout the region of interest ROI_sim_. Finally, this process was repeated for a variety of spoiling moments I_spoil_xT_spoil_ and for several potential crusher coil designs.

### Subjects and Materials

The eight subjects (males, BMI 21–25 kg·m^−2^) enrolled in this study gave informed consent according to the procedure approved by the local Research Ethics Committee. A 7T actively shielded whole‐body MRI scanner (Siemens, Erlangen, Germany) was used for all scans. Localization was performed using a separate 10 cm ^1^H Transmit/Receive (Tx/Rx) loop coil (Rapid Biomedical, Germany) to acquire cardiac‐gated CINE FLASH images. ^31^P‐MR spectra were acquired with a 10 cm ^31^P Tx/Rx loop. The crusher coil was placed between the phantom/volunteer and the ^31^P Tx/Rx coil.

Three phantoms were used for this study: (i) phantom A: a cuboid (40 × 40 × 50 mm^3^) containing KH_2_PO_4_ immersed in a 4 L cuboid (290 × 85 × 220 mm^3^) containing 73 mM NaCl; (ii) phantom B: an acrylic cuboid (120 × 170 × 30 mm^3^) containing 0.2 M H_3_PO_4_ (δ_H3PO4_ = −1.2 ppm); and (iii) phantom C: an acrylic cuboid (200 × 330 × 30 mm^3^) containing 0.2 M K_2_HPO_4_^2‐^ (δ_K2HPO4_ = 1.4 ppm).

### Crusher Coil

The crusher coil was built following a “meander” design first proposed by Wiesler et al 19, 24. To reduce the overall current required for spoiling, we elected to use bunches of four linear current‐bearing wires for each leg of the “meander”. The “meander” shape (i.e., alternating anti‐parallel current directions) is designed to produce a high **B**_spoil_ gradient near the coil, which decreases rapidly away from the coil, as previously reported 22. Current return paths were compensated to minimize the torque created when spoiling. A single piece of insulated copper wire (1 mm diameter) was used to construct the entire meander. This wire was mounted in grooves routed into a UPVC panel (300 × 220 × 4 mm^3^). The conducting paths in the meander coil all lay within a 280 × 200 mm^2^ region. Inductive low pass filters (240 nH) were placed every λ/10 (∼250 mm) by winding the single piece of wire into a helix at the appropriate points. These inductors served to isolate the crusher coil from the Tx/Rx coil. To prevent exposure of the subject to the DC voltage applied to the crusher coil and to provide mechanical rigidity, the wire was glued into the UPVC panel and covered by another plastic board, giving a finished crusher coil with a total thickness of 15 mm. This covering further separates the crusher coil from the RF coil, which minimizes any remaining chance for capacitive or inductive coupling with the Tx/Rx coil.

A reservoir capacitor, continuously charged by a power supply unit (PSU, Iso‐Tech IPS23002A), was used to drive the high (I_spoil_ up to 40A), short (T_spoil_ = 100–200 μs) DC pulses in the crusher coil (i.e., I_spoil_ × T_spoil_ < 8 A·ms). Further details about the circuitry and the overall setup are provided in the Supporting Information (Fig. S1), which is available online. To verify that there was negligible interaction between the RF coil and the crusher coil, a transmit RF field (B_1_^+^) mapping experiment was performed (see Supporting Information for details, Fig. S2).

RF heating safety tests confirmed that the combination of crusher coil and RF coil together produced no more RF heating than the RF coil alone (see Supporting Information for details, Fig. S3).

A T_spoil_ = 100 μs DC pulse was applied to the crusher coil immediately after the RF excitation pulse in the dead‐time already used for phase encoding gradients 8. In these experiments, we cautiously also added a further 50 µs delay to be sure that the crusher coil had also rung down before data acquisition, giving an overall dead‐time of 150 μs between end of RF pulse and start of acquisition.

### Experiment 1: Preliminary Spoiling Demonstration

To calibrate the level of intra‐voxel dephasing from the crusher coil, a preliminary experiment was performed in vitro using a pulse‐acquire sequence [repetition time (TR) = 1000 ms, echo time (TE) = 0.85 ms, bandwidth (BW) = 6000 Hz], on a two‐compartment phantom assembly: phantom A (100 mm from the RF coil), with phantom B above that (20 mm from the RF coil) and the RF coil on top. Excitation was with a hard pulse having a flip angle of ∼7° in phantom A and 20°–50° across phantom B. The crusher coil was placed on phantom B, and the ^31^P RF coil was placed on the crusher coil. These conditions were chosen to model the in vivo geometry, i.e., phantom B corresponds to “skeletal muscle” and phantom A corresponds to “myocardium.” To determine the optimum spoiling current before the in vivo study, I_spoil_ × T_spoil_ was increased in steps from 0 to 2.4 A·ms and the corresponding phantom B signal residual simulations were compared with experimental values using similar conditions (V_sim_ size and depth).

### Experiment 2: Validation of The Spoiling Field Simulations

To validate our simulations, a three‐dimensional (3D) ultrashort echo time chemical shift imaging (UTE‐CSI) experiment was performed with phantom B (in the sagittal plane) with the following parameters: the UTE‐CSI sequence in Rodgers et al 8, TR = 1000 ms, TE = 0.85 ms, BW = 8000 Hz, over a transverse 10 × 10 × 8 matrix, over a 200 × 200 × 160 mm^3^ FOV, using acquisition weighting with 24 averages at k = 0, and I_spoil_ × T_spoil_ was increased in steps from 0.85 to 4.25 A·ms. The flip angle varied from 80° at the top of the phantom to 5° at the bottom. A similar setup was used to compare the SNR with and without the crusher coil in place (see Supporting Information for details, Fig. S4).

### Experiment 3: Phantom Comparison of BISTRO versus Crusher Coil Saturation

To compare the residual signal when using the crusher coil and/or BISTRO saturation band, a 2D UTE‐CSI experiment was performed with a two‐slice phantom, consisting of phantom B resting on phantom C. This experiment was run with the following acquisition parameters: TR = 1000 ms, TE = 0.85 ms, BW = 8000 Hz, over a transverse 12 × 12 matrix with a slice thickness = 20 mm, over a 180 × 180 mm^2^ FOV, using acquisition weighting with 24 averages at k = 0. Sinc pulse was used for excitation giving a flip angle of approximately 20°–50° across phantom B and 5°–15° across phantom C. Four different approaches to eliminate “contaminating” signal from the top slice were investigated: (i) standard offer versus serve (OVS) saturation (sinc pulse with bandwidth 1000 Hz and pulse length 7680 μs, SAR = 82%), (ii) BISTRO saturation [details in Rodgers et al 8, SAR = 96%], (iii) crusher coil (I_spoil_ × T_spoil_ = 3.2 A·ms, SAR = 16%), and (iv) BISTRO saturation and crusher coil (I_spoil_ × T_spoil_= 2.2 A·ms, SAR = 96%). The saturation band covered the top slice (band thickness = 30 mm). Only voxels entirely covered by the saturation band were used for the calculation of the residual signal in phantom B.

### Experiment 4: In Vivo Comparison of BISTRO versus Crusher Coil Saturation

To investigate the use of the crusher coil in vivo, a 3D UTE‐CSI experiment was performed with the following acquisition parameters: TR = 1000 ms, TE = 0.85 ms, BW = 8000 Hz, over a transverse 16 × 16 × 8 matrix, covering a 240 × 240 × 200 mm^3^ FOV, using acquisition weighting with 10 averages at k = 0, giving acquisition time = 28 min, nominal voxel size = 15 × 15 × 25 mm^3^ and true voxel size = 57.8 mL (i.e., this is the volume within 50% of peak point spread function). RF excitation was always at the full power supported by the coil (270 V) giving a flip angle of ∼50° in the skeletal muscle and ∼20° in the interventricular septum. BISTRO saturation band was placed in the coronal plane to suppress skeletal muscle signal (band thickness = 25 mm). The BISTRO saturation voltage was maximized for each study subject to the SAR limit. Typically, a 70 V peak voltage for BISTRO gave 96 ± 1% SAR overall for protocols including BISTRO saturation. In contrast, the SAR was only 16 ± 1% when the crusher coil was used without BISTRO.

Three in vivo protocols were used for the main study: (i) no signal suppression (i.e., neither BISTRO, nor crusher coil), (ii) BISTRO saturation, (iii) crusher coil (I_spoil_ × T_spoil_ = 0.9 A·ms).

As this study was just under the maximum permitted scan duration under institutional rules (i.e., 2 h), this full three‐protocol comparison was performed in 2 subjects and used to compare the skeletal muscle signal suppression. Additional data was then collected with either protocols (i) versus (ii) or (i) versus (iii) to further compare the quantification reliability of the crusher coil against RF‐based saturation (n = 4).

We also separately tested: (iv) *both* crusher coil (I_spoil_×T_spoil_ = 1 A·ms) spoiling *and* BISTRO saturation in an additional subject, to further compare skeletal muscle signal suppression; and (v) crusher coil with the following scans in one session: I_spoil_ × T_spoil_ = 0.0 A·ms, I_spoil_ × T_spoil_ = 0.9 A·ms, I_spoil_ × T_spoil_ = 2.0 A·ms and I_spoil_ × T_spoil_ = 3.0 A·ms, in two further subjects, to test the effects of increased levels of spoiling. For these scans, we chose a TR of 800 ms to remain within an acceptable overall scan time.

### Experiment 5: Demonstration of SAR‐demanding Sequence Using Crusher Coil

To demonstrate the potential of the crusher coil for SAR‐intensive studies in the heart at 7T, we performed frequency‐selective saturation with a delays alternating with nutations for tailored excitations (DANTE) pulse train 26, 27. At 7T, all the available SAR is required to achieve reliable saturation in the heart of a metabolite (e.g., γ‐ATP). With RF‐based saturation, one must, therefore, compromise between an acceptable suppression of skeletal muscle contamination and acceptable γ‐ATP saturation. Yet, with the crusher coil, no such compromise is required. Specifically, we used a DANTE train with subpulse duration T_p_ = 100 μs, subpulse TR_p_ = 330 μs and fixed subpulse amplitude. This DANTE train was inserted during the period following each readout, starting after the readout and ending before the next excitation in the 3D UTE‐CSI sequence used above. The crusher coil (I_spoil_ × T_spoil_ = 0.9 A·ms) was used to suppress the overlying skeletal muscle signal. The γ‐ATP peak was saturated and control saturation was performed with a symmetric DANTE excitation frequency on the left of PCr.

### Data Analysis

Spectra from the myocardium and from overlying skeletal muscle were analyzed with a custom Matlab implementation 29 of AMARES (advanced method for accurate, robust, and efficient spectral fitting) 30. Prior knowledge specifying 11 Lorentzian peaks (PCr, α,β,γ‐ATP, PDE, and 2,3‐DPG) with fixed amplitude ratios and scalar couplings for the multiplets was provided. Correction for blood contamination and partial saturation, calculation of SNR and calculation of Cramer‐Ráo lower bounds (CRLB) followed Rodgers et al 8. The skeletal muscle residual signal was measured using the mean residual PCr amplitude in skeletal muscle voxels overlying the heart. Cardiac ^31^P spectra were measured in the interventricular septum. Results (PCr/ATP ratio, CRLB of the ratio, SNR and linewidth of PCr) were averaged for “no saturation” case (n = 6), BISTRO saturation (n = 4), and for the crusher coil at I_spoil_ × T_spoil_ = 0.9 A·ms (n = 6) and at I_spoil_ × T_spoil_ = 2 A·ms (n = 2). Note that the spoiling is reported in units of A·ms because the high inductance of the meander coil led to appreciable, but reproducible, rise‐ and fall‐times for the DC pulse (see Supporting Information, Fig. S2). In practice, the pulse timing was fixed and the PSU voltage (and hence I_spoil_) was varied.

In phantoms and in vivo, the residual signal S_r,exp_ was obtained by normalizing, for each voxel in turn, the signal S_i_ after signal suppression (i.e., with BISTRO and/or the crusher coil) relative to the unsuppressed signal S_0_:

(4) $$S_{r,\exp}=\frac{S_{i}}{S_{0}}\times 100\%$$

SAR results are reported as percentage of the “Normal controlled mode” for a local transmit coil in the trunk, defined by the IEC guidelines 17 (i.e., 100% SAR corresponds to SAR = 10 W/kg).

## RESULTS

### Simulations of Spoiling Field and the Ensuing Signal Suppression

**Figure 1:**
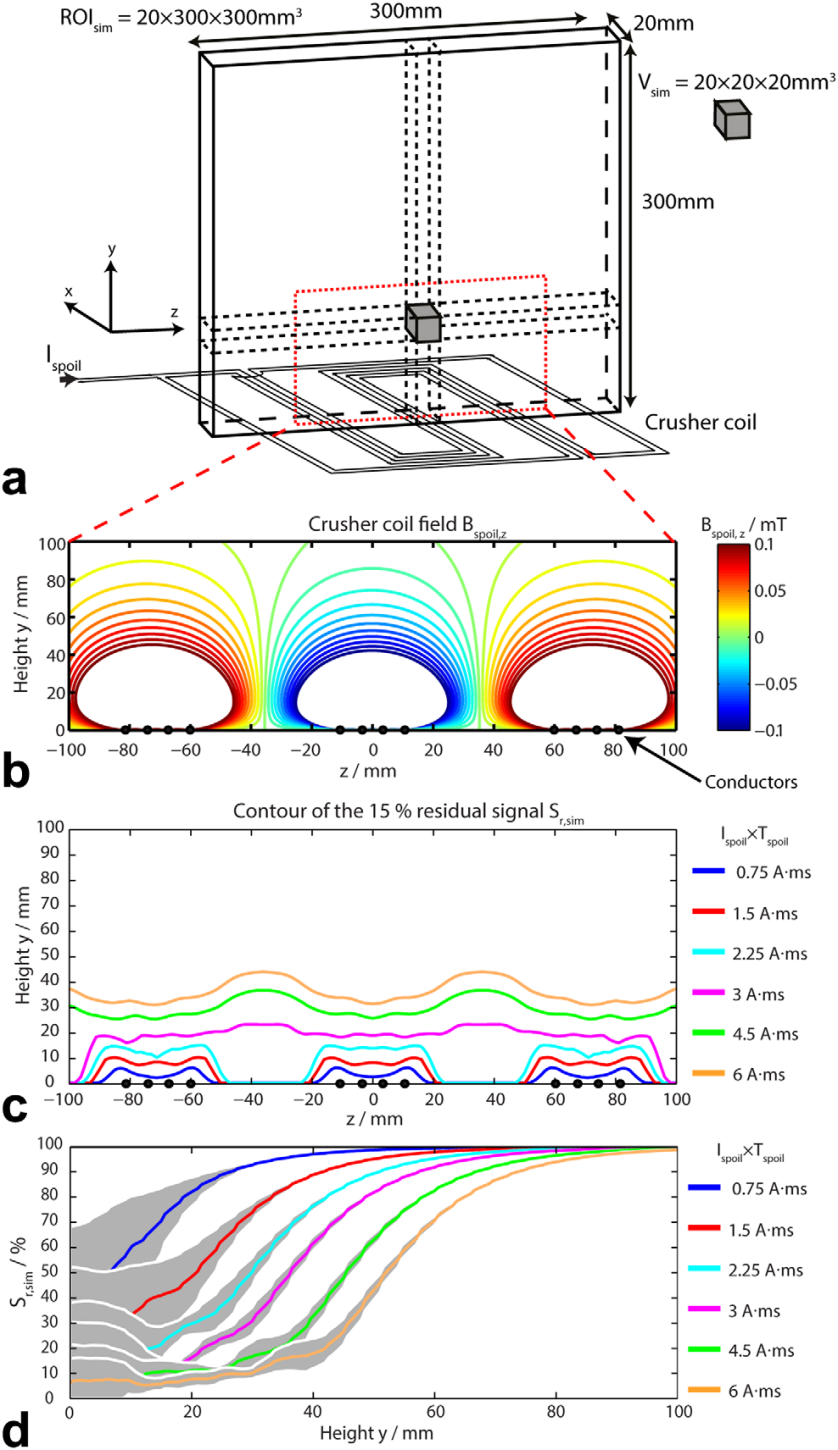
**A**: The simulated region of interest (ROI_sim_) was averaged over 20 × 20 × 20 mm^3^ simulated voxels of interest V_sim_. **B:** Calculated crusher coil field B_spoil,z_ with I_spoil_ = 10 A for the crusher coil geometry shown in (A). **C:** Contour plot of the 15% residual signal S_r,sim_ above the crusher coil for different I_spoil_×T_spoil_. **D:** Simulated residual signal S_r,sim_ as a function of the height above the crusher coil for different I_spoil_×T_spoil_. For each I_spoil_×T_spoil_, the residual signal was averaged over the region −100 mm < z < 100 mm. The data of plot (D) are represented as intervoxel mean (solid line) ± intervoxel standard deviation (SD) (gray shade). The intervoxel variability is calculated at a specific height y. (B), (C), and (D) plots are zoomed to show only the region bounded by the red dashed line in (A).

Following the study by Wiesler et al 19, we performed |**B_spoil_**| simulations (Figs. 1A,B), which were in agreement. Then, considering our setup and the change in gyromagnetic ratios (i.e., γ_31P_<γ_1H_), we computed corresponding residual signals (Figs. 1C,D). The length of the wires (l_x_), the number of wires, the spacing between the wires (G), and the spacing between the meanders (D) were chosen to yield minimum residual signal at depths < 40 mm (i.e., skeletal muscle) and limited signal suppression at depths >70 mm (i.e., the heart) (Figs. 1C,D). From our simulations, we observed that a range of spacings between meanders (40 mm < D < 70 mm) and between wires (6 mm < G < 9 mm) all gave acceptable performance for these depth ranges. From these, we chose the spacings (D = 70 mm and G = 7 mm) most convenient for use with our coil geometry.

Note, however, that the optimum spacing for other applications may change. If the protocol needs very spatially homogeneous signal suppression in the plane parallel to the crusher coil, this would require a smaller spacing G between meanders; if the protocol needs to suppress signal over a thicker slab, this would require a larger spacing D between wires; or if the protocol has larger voxels, the signal saturation will occur for lower I_spoil_ × T_spoil_, and thus the number of wires in each bunch could be decreased.

With weak spoiling (I_spoil_ × T_spoil_ < 2.25 A·ms), residual signal close to the crusher coil was relatively inhomogeneous (Fig. 1C). This is because the crusher gradient is stronger above the wires, and so leaves a low residual signal, but it is weaker between the wires, and therefore leaves a higher residual signal there. With strong spoiling (I_spoil_ × T_spoil_ > 2.25 A·ms), these residual signal inhomogeneities were less noticeable. This effect is shown clearly by the residual signal standard deviations (SD) plotted in Figure 1D. With weak spoiling, the residual signal SD was almost 30% near the crusher coil, but it was <10% for strong spoiling.

Note the transition band from 20% to 90% residual signal at a depth of 40–70 mm in Figure 1C. This suggests that a crusher coil with our design can suppress contaminating tissue without affecting the region of interest, providing the contaminating tissue is more than 30 mm from the region of interest. Considering these simulations and the typical voxel size in a ^31^P‐MRS experiment, we estimated that I_spoil_ and T_spoil_ would never need to exceed 40 A and 200 μs, respectively (i.e., I_spoil_ × T_spoil_ < 8 A·ms).

**Figure 2:**
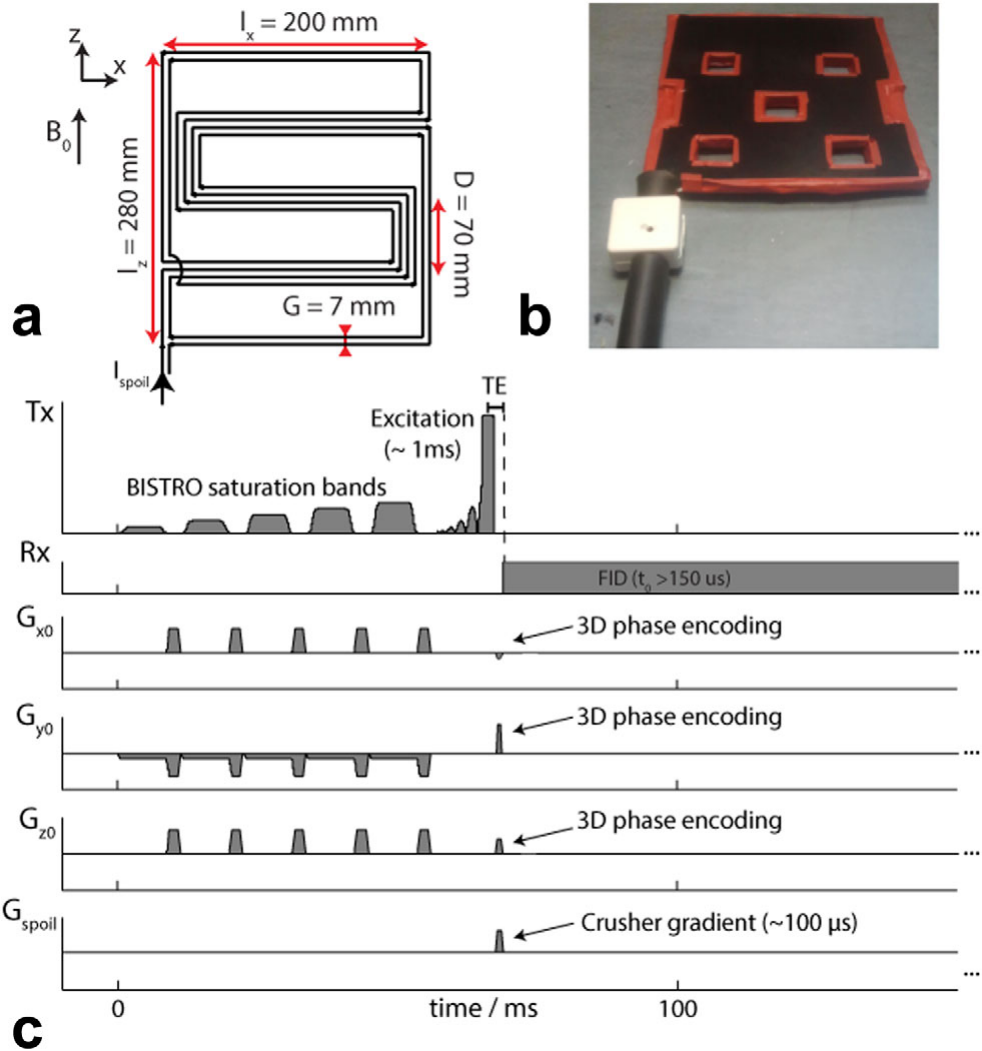
**A**: Schematic of the crusher coil with the optimized dimensions. **B:** Photograph of the assembled crusher coil. **C:** Sequence diagram of the 3D‐CSI sequence used to acquire ^31^P MR spectra. Note that the crusher coil is pulsed simultaneously with the phase encoding gradients. The RF excitation pulse has been amplified in the illustration for clarity.

The crusher coil was built as illustrated in Figures 2A,B. The acoustic noise from the pulsing crusher coil was far less than from standard imaging sequences and therefore poses no danger to subjects. The crusher gradient pulse was timed to coincide with the phase encoding gradients in the 3D‐CSI sequence, as shown in Figure 2C.

We used a point source phantom positioned 40 mm below the surface of the crusher coil (see Supporting Figure S3) and measured the RF field by a series of pulse‐acquire spectra with a range of excitation pulse voltages. The B_1_^+^ was 0.046 μT/V with the RF coil and the crusher coil in place, 0.053 μT/V with the RF coil alone (crusher coil replaced by padding) and 0.062 μT/V with the RF coil on top of the phantom (without padding).

The SNR was measured from a 3D‐CSI acquisition in phantom B (see Supporting Figure S4), with and without the crusher coil. The SNR slightly decreased over the slice by 13 ± 5% when the crusher coil was inserted.

### Experiment 1: Preliminary Spoiling Demonstration

**Figure 3:**
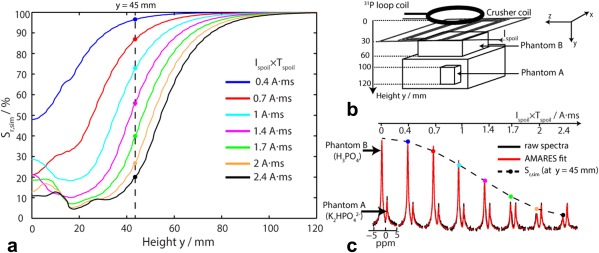
**A**: Mean simulated residual signal S_r,sim_ as a function of height above the crusher coil for different spoiling moments I_spoil_×T_spoil_. **B:** Schematic diagram of the two‐compartment phantom assembly, crusher coil and ^31^P 10 cm loop RF coil (experiment 1). **C:** Nonlocalized spectra acquired from a two‐compartment phantom (raw spectra in black, AMARES fits in red) for various spoiling moments (I_spoil_ × T_spoil_ = 0 to 2.4 A·ms). Signals from the phantom B (i.e., proximal to the crusher coil, containing H_3_PO_4_, right peak) were suppressed, but signals from phantom A (i.e., far from the crusher coil, containing K_2_HPO_4_^2‐^, left peak) remained unaffected. The dashed line corresponds to the S _r,sim_ of (A) at 45 mm depth, which corresponds to the center of phantom B.

FIDs were acquired from the two‐compartment phantom (Fig. 3B) with a series of different values for I_spoil_ × T_spoil_. The signal from phantom B (i.e., close to the crusher coil) started to be spoiled at I_spoil_ × T_spoil_ = 1 A·ms and was reduced to <20% of its initial value at I_spoil_ × T_spoil_ = 2.4 A·ms. Meanwhile, the signal from phantom A (i.e., far from the crusher coil) was still > 80% of its initial value at I_spoil_ × T_spoil_ = 2.4 A·ms. These experimental values were compared with simulation using similar conditions.

### Experiment 2: Validation of the Spoiling Field Simulations

**Figure 4:**
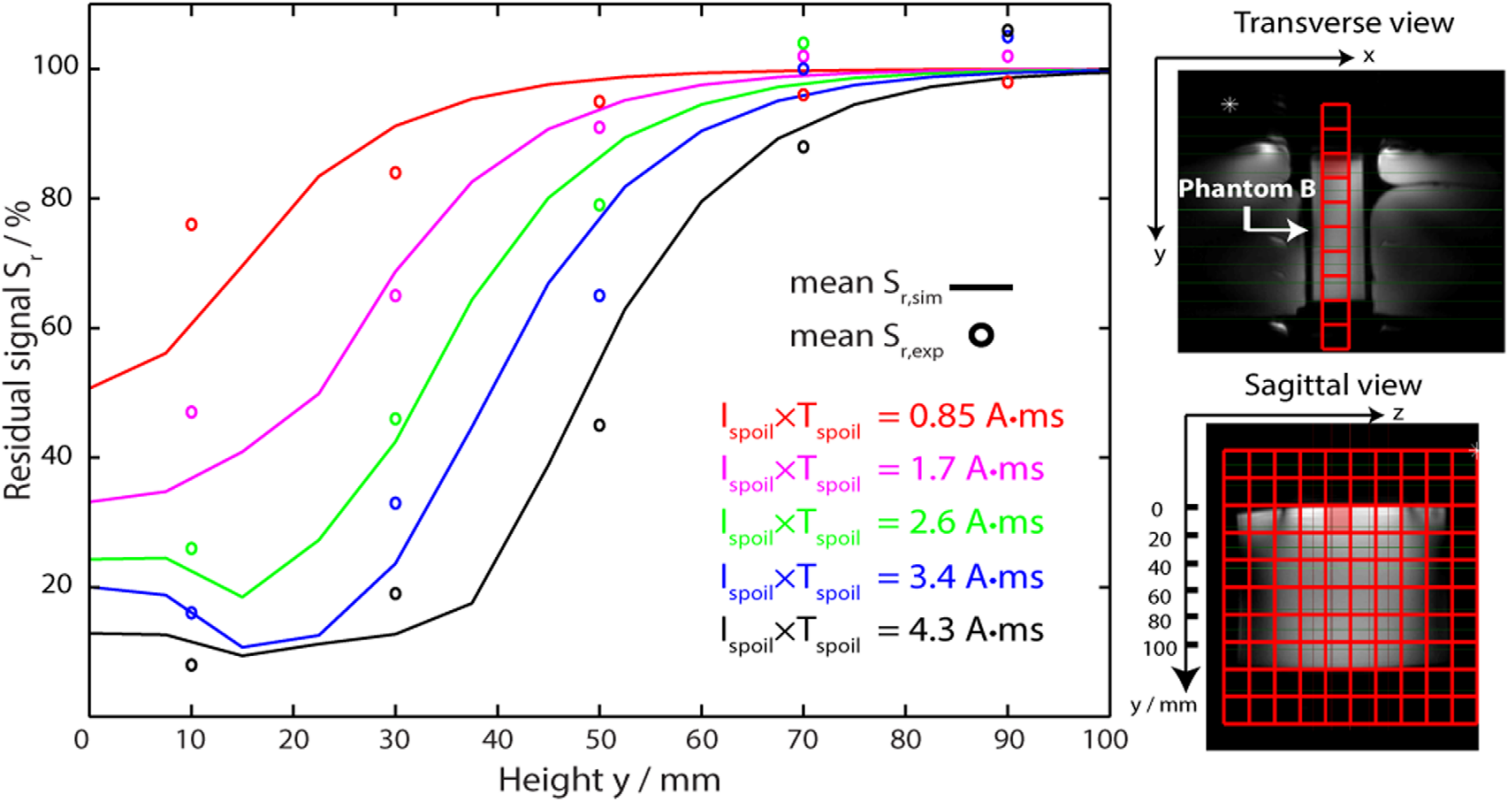
Residual signals were simulated S_r,sim_ (solid lines) and measured S_r,exp_ (circles) in phantom B as a function of height (experiment 2). The spoiling moment I_spoil_ × T_spoil_ was kept constant when comparing experimental data and simulations. The simulations used the same position of the CSI grid relative to the crusher coil as the experiments. Insets: CSI matrix plotted over ^1^H FLASH images. Phantom B was placed between two saline bags to load the coil.

Figure 4 shows the extent of signal spoiling measured by 3D‐CSI as a function of depth for different I_spoil_ × T_spoil_ in a phantom. Experimental data points were all within one SD of the simulation mean values. Indeed, all the experimental data points lay within 10% of the simulation mean values, except those with I_spoil_ × T_spoil_ = 0.85 and 1.7 A·ms at a depth of 10 mm which lay within 20%. The agreement between simulation and experiment demonstrates that our simulations will be sufficient to set an appropriate I_spoil_ and T_spoil_ to suppress signals at a prescribed depth in vivo.

### Experiment 3: Phantom Comparison of BISTRO versus Crusher Coil Saturation

**Figure 5:**
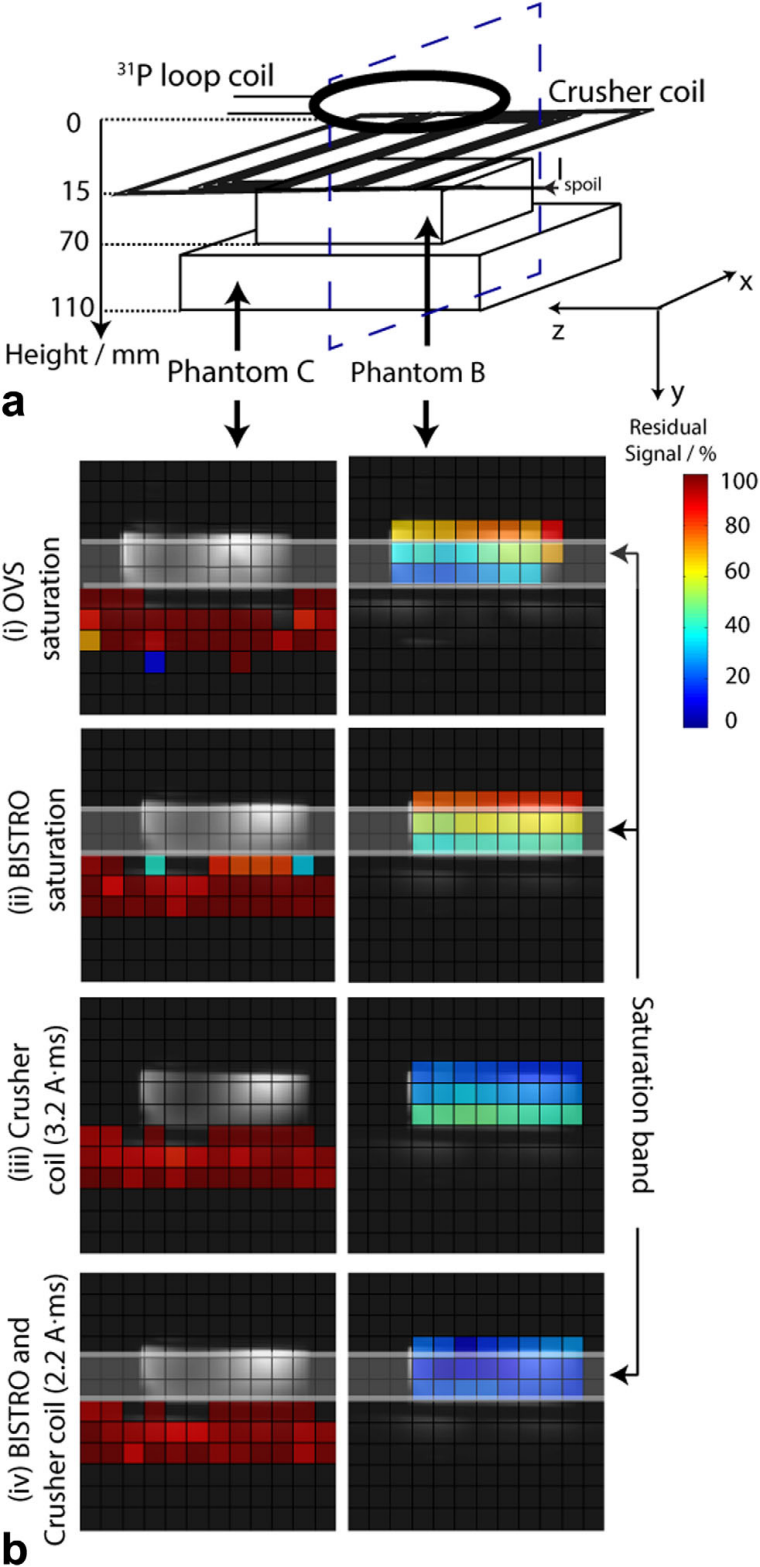
**A**: Schematic of the experimental setup (experiment 3), showing the two‐slice phantom containing two different ^31^P compounds: H_3_PO_4_ (Phantom B) and K_2_HPO_4_^2‐^ (Phantom C). **B:** Peak amplitudes fitted from the ^31^P CSI spectra from a transverse slice, (blue dashed line in (A)), normalized to the amplitudes without any signal suppression technique. Phantom B signal (i.e., top slice) is shown on the right and Phantom C signal (i.e., bottom slice) is shown on the left. The different signal suppression schemes used were: (i) OVS saturation, (ii) BISTRO saturation; (iii) crusher coil (I_spoil_ × T_spoil_ = 3.2 A·ms); (iv) crusher coil (I_spoil_ × T_spoil_ = 2.2 A·ms) and BISTRO. The prescribed BISTRO saturation band is marked as a semi‐transparent white band (band thickness = 30 mm). Background voxels with intensity <30% of the maximum were excluded for clarity.

Four signal suppression methods, detailed in the Methods, were compared in a 2D‐CSI phantom experiment (Fig. 5). Phantom experiments were performed using the same voltage limitations as applied in vivo. The mean signal amplitudes of the two phantoms are shown in Table 1 for the different cases. The bottom phantom signal varied by <5% in all cases, while the top phantom signal was reduced to 15–50% of its unsuppressed value depending on the acquisition protocol. Standard OVS saturation and BISTRO saturation gave a 35 ± 13% and 50 ± 10% residual signal in the top phantom, respectively. The crusher coil gave a lower residual signal of 34 ± 8% (with I_spoil_ × T_spoil_ = 3.2 A·ms). When using BISTRO and crusher coil together at the same time, the mean top phantom signal was further reduced to 15 ± 5% of its initial value.

**Table 1:** Comparison of Residual Signal Using OVS Saturation, BISTRO, and Crusher Coil in the Two‐Slice Phantom (Experiment 3)^a^

	OVS saturation	BISTRO	Crusher coil	Crusher coil and BISTRO
S_r,exp_ /% (top slice)	35 ± 13	50 ± 10	34 ± 8	15 ± 5
S_r,exp_ /% (bottom slice)	100 ± 10	95 ± 10	96 ± 8	96 ± 6

a Residual signals S_r,exp_ in the two‐slice phantom with four different 2D‐CSI in vitro acquisition protocols: (i) OVS saturation, (ii) BISTRO saturation, (iii) crusher coil (I_spoil_×T_spoil_= 3.2 A·ms), (iv) crusher coil (I_spoil_×T_spoil_= 2.2 A·ms) and BISTRO. Percentage residual signals are given as mean ± SD.

Although, the residual signal comparison with standard OVS saturation is of academic interest, practically, it must be remembered that this method cannot be used for ^31^P‐MRS in the human heart because of severe chemical shift displacement artefacts that would obliterate important signals in the myocardium when saturating phosphocreatine in skeletal muscle, and because of the sensitivity to B_1_^+^ inhomogeneity in vivo. These factors are discussed in detail in the Supporting Information of Rodgers et al 8.

### Experiment 4: In Vivo Comparison of BISTRO Versus Crusher Coil Saturation

**Figure 6:**
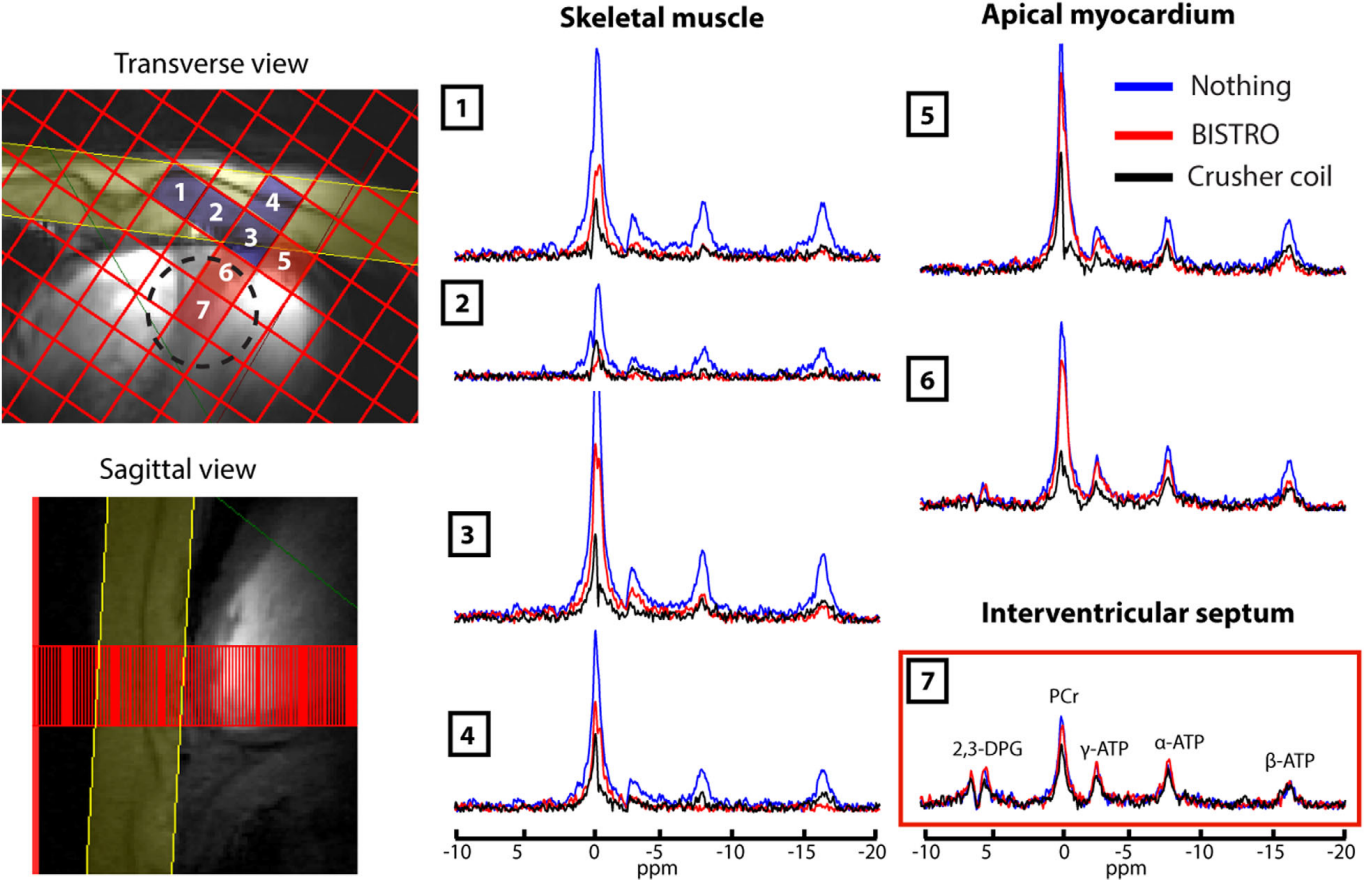
The 3D‐CSI cardiac spectra acquired with three suppression protocols: no saturation (blue spectra); BISTRO saturation (red spectra); and using the crusher coil with I_spoil_ × T_spoil_ = 0.9 A·ms (black spectra) (experiment 4). Left: CSI matrix overlaid on 7T CINE FLASH localizers showing the locations of voxels 1–7 and of the BISTRO saturation band (in yellow). Voxels are shown that contain primarily skeletal muscle (blue) or myocardium (red). Voxel 7 is representative of the interventricular septum and was quantified with AMARES as shown in Table 2. The dashed circle denotes the 50% of maximum point spread function contour for voxel 7 (i.e., the “true” voxel size).

The 3D‐CSI was then performed in vivo (Fig. 6) with the three signal suppression protocols described in the Methods. The mean PCr signal in skeletal muscle in the voxels overlying the heart (e.g., voxels 1–4 in Figure 6) was reduced to 49 ± 4% (mean ± intersubject SD) of its unsuppressed value by BISTRO and it was reduced to 36 ± 1% by the crusher coil. These results were stable and reproducible from run‐to‐run. In further experiments (not shown), where the crusher coil was used together with a BISTRO saturation band, the mean PCr signal in skeletal muscle was slightly lower at 21 ± 5% of its initial value.

Table 2 shows the quantitative parameters computed from ^31^P spectra acquired in the interventricular septum (e.g., voxels 7 in Figure 6). The PCr/ATP ratio was 2.4 ± 0.6 without any saturation scheme, which is somewhat higher than the accepted normal range of 1.5–2.2 31 and shows that there is skeletal muscle contamination of this “septal” voxel. The PCr/ATP ratio falls to acceptable values of 2.1 ± 0.4 when saturating with BISTRO, and 1.8 ± 0.3 when using the crusher coil (I_spoil_ × T_spoil_ = 0.9 A·ms). The PCr/ATP CRLB and PCr linewidth were similar between these three protocols.

**Table 2:** Cardiac ^31^P Spectral Metrics from the Interventricular Septum for Three Skeletal Muscle Suppression Strategies (Experiment 4)^a^

	No saturation	BISTRO	Crusher coil	Crusher coil
Number of subjects	6	4	6	2
Spoiling moment I_spoil_×T_spoil_ / A·ms	‐	‐	0.9	2
Blood‐ and saturation‐corrected PCr/ATP ratio	2.4 ± 0.6	2.1 ± 0.4	1.8 ± 0.3	1.8 ± 0.4
PCr/ATP CRLB /%	17 ± 5	19 ± 9	22 ± 3	31 ± 8
PCr linewidth / Hz	26 ± 3	27 ± 4	30 ± 8	32 ± 10
SAR /%	16 ± 1	96 ± 1	16 ± 1	16 ± 1

Number of subjects, spoiling moment (I_spoil_×T_spoil_), blood‐ and saturation‐corrected PCr/ATP ratio, CRLB of PCr/ATP ratio, linewidth (lw_PCr_) of PCr peak, and SAR for four different 3D‐CSI in vivo acquisition protocols: (i) no saturation (ii) BISTRO saturation, (iii) crusher coil with I_spoil_×T_spoil_ = 0.9 A·ms, and (iv) I_spoil_×T_spoil_ = 2 A·ms. Data are reported as mean ± inter‐subject SD. PCr/ATP ratio and CRLB_PCr/ATP_ were statistically significant (p < 0.05, two‐tailed unpaired t‐test) only between (i) no saturation and (iii) crusher coil (I_spoil_×T_spoil_ = 0.9 A·ms). Note that TR was 800ms for protocol (iv), and TR was 1000ms for the other protocols.

When higher saturation protocols were used, the mean PCr signal in skeletal muscle was reduced to 24 ± 5% (I_spoil_ × T_spoil_ = 2 A·ms) and 12 ± 1% (I_spoil_ × T_spoil_ = 3 A·ms). However, accurate quantification of the interventricular septum voxel was only possible with I_spoil_ × T_spoil_ = 2 A·ms at the edge of quantification reliability (PCr/ATP ratio = 1.8 ± 0.3 and CRLB_PCr/ATP_ = 31 ± 8%) (Table 2).

### Experiment 5: Demonstration of SAR‐demanding Sequence Using Crusher Coil

**Figure 7:**
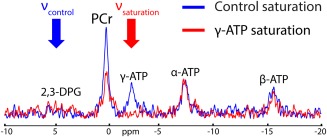
^31^P‐MR spectra acquired with 3D‐CSI in the interventricular septum (experiment 5) with DANTE control saturation at ν_control_ (blue spectrum) or with DANTE saturation of γ‐ATP at ν_saturation_ (red spectrum).

A γ‐ATP frequency‐selective saturation study was performed with the use of a crusher coil to saturate the myocardial skeletal muscle signal (Fig. 7). The irradiation of DANTE subpulses at γ‐ATP frequency led to the complete suppression of the γ‐ATP spectral peak.

## DISCUSSION

### Validation of Crusher Coil Spoiling Depth

The crusher coil geometry was designed to give minimal residual signal at depths < 40 mm and limited signal suppression at depths > 70 mm, subject to a maximum current I_spoil_ < 40 A (due to the PSU, switching circuit and crusher coil resistance) and a maximum spoiling time T_spoil_ < 100 μs by means of simulations (Figs. 1, 2).

The agreement between simulations and experimental data (Figs. 3, 4) confirms that we can use the simulations to set I_spoil_ and T_spoil_ to spoil to a desired depth. This was further confirmed as the spoiling moment I_spoil_ × T_spoil_ was in the same range as a previous ^31^P‐MRS liver study at 2T 23. While the degree of signal suppression from BISTRO saturation bands is limited by the available peak B_1_^+^, with the crusher coil, we could set I_spoil_ up to 40 A, and, therefore, we could choose the degree of desired suppression in skeletal muscle balancing it against unwanted suppression in the myocardium. Similarly, T_spoil_ can easily be altered providing it fits within the existing pulse sequence dead‐time. This flexibility of the crusher coil means that one could adapt the signal suppression depth to suit a particular subject's anatomy, which is expected to be particularly important for female, obese or very muscular volunteers.

### Crusher Coil for Cardiac ^31^P‐MRS

No subject discomfort was reported during crusher coil scans. Crusher coil placement was straightforward. The I_spoil_ × T_spoil_ required to suppress efficiently skeletal muscle for each subject was between 0.8 and 1.2 A·ms. So, in practice a single value of 1.0 A·ms could have been used for all in vivo scans without compromising skeletal muscle signal suppression, illustrating the ease‐of‐use of the crusher coil. To maximize signal suppression in vivo, the wires of the crusher coil were placed so wires ran directly over the volume of interest.

Given B_0_ shimming limitations and B_1_ variations in the skeletal muscle at 7T, RF saturation bands must be designed to robustly suppress unwanted signals. However, at ultra‐high field, the maximum peak B_1_^+^, the length and the number of saturation train pulses are limited. Together, these factors mean that only incomplete saturation can be achieved 8. To overcome this limitation, this study introduced a crusher coil to suppress these undesired signals instead of (or in addition to) using RF saturation bands. The signal saturation efficiency of the crusher coil compared with BISTRO was demonstrated in phantom studies (Figure 5 and Table 1) and for cardiac ^31^P in vivo studies (Figure 6 and Table 2). Improved signal saturation was obtained when using simultaneously the crusher coil and BISTRO.

The AMARES fitting results (Table 2) reported similar spectra quantification acquired in the interventricular septum, independent of whether BISTRO saturation or the crusher coil was used. The quantification precision (i.e., CRLB) and linewidth were similar between all the cases. A small PCr/ATP decrease was observed when using the crusher coil, but of course this could be due to lower skeletal muscle contamination. The anomalously high PCr/ATP ratio in the “no saturation” case clearly illustrates the need for skeletal muscle suppression when performing 3D‐CSI in the human heart. Spectral quality in the presence of the crusher coil was good, e.g., the linewidth of PCr was 30 ± 8 Hz here, compared with 37 ± 11 Hz in our previous 7T cardiac ^31^P MRS study 8.

It is possible to saturate skeletal muscle to give residual less than 12% (I_spoil_ × T_spoil_ = 3 A·ms). However, given the limited FOV of the 10 cm ^31^P loop used here, the precision of spectral quantification in the deepest regions detectable by the coil would be compromised. To acquire a good ^31^P MR spectrum from the heart without contamination of signals from skeletal muscle, a I_spoil_ × T_spoil_ higher than 2 A.ms may be required, thus necessitating receive coils with a deeper field on view and additional SNR. Increasing I_spoil_ × T_spoil_ could also be relevant for different volunteers (e.g., women or obese volunteers).

### Possibilities Afforded by the Crusher Coil

The crusher coil is an efficient alternative to BISTRO saturation bands for pulse sequences that are limited by SAR. For instance, the TR of a pulse sequence may be drastically reduced when using a crusher coil, as previously reported for ^1^H‐MRS in the human brain 20. Importantly, this also opens the possibility to implement more SAR‐demanding pulse sequences in the heart at 7T, without compromising on the suppression of unwanted (potentially contaminating) signals from overlying skeletal muscle. To demonstrate this potential, we performed a final experiment using a DANTE selective saturation pulse sequence 26, 27, 32 in conjunction with the crusher coil to robustly saturate the myocardial skeletal muscle signal (Fig. 7).

The use of a crusher coil was previously demonstrated for rat liver spectroscopy 21. There is a growing interest in characterizing the human liver by MR 33, 34, 35, 36. This crusher coil could also be used for human liver ^31^P MRS studies at 7T, which have similar challenges of muscle tissue contamination and SAR‐limited pulse sequences to overcome 33. Finally, it is important to emphasize that while we have demonstrated the crusher coil using BISTRO and nonlocalized or CSI‐localized spectroscopy, the crusher coil can be used in conjunction with any localization approach that has a ∼150 µs dead time according to the needs of the study that is envisaged.

### Limitations of the Crusher Coil

Recently, Boer et al 20 reported the use of a crusher coil for human brain ^1^H‐MRS studies to remove extracerebral lipid signal from the outer volume. They reported results for single slice CSI, noting that it might be necessary to choose I_spoil_ to give the correct signal suppression for CSI over a particular slice. This is similar to our conclusions from the simulations shown in Figure 1.

To determine the residual signal in the skeletal muscle and its potential impact on quantification of interventricular septum spectra, the CSI grid was placed in the transverse plane for these in vivo studies. This was done to establish the proof‐of‐principle of the crusher coil for cardiac ^31^P‐MRS at 7T. However, now the signal suppression delivered by the crusher coil is understood, there is no reason not to run with any desired CSI orientation.

If the pulse sequence and RF coil used in a study happens to give ∼180° excitation in the skeletal muscle, there would be negligible transverse magnetization there, and so there would be negligible contaminating signal in the resulting spectra (without using the crusher coil). However, in our cardiac studies, the range of flip angles in the skeletal muscle is 50° to 80°, so there is very efficient spoiling.

Note that the transition band (20–30 mm, where intermediate spoiling occurs) may be an issue if the suppressed region and the region of interest are in close proximity. The sharpness of the transition between spoiling and not‐spoiling regions depends on the wire spacing in the crusher coil. Alternatively, simulations suggest that in many cases placing the crusher coil further away from the subject (i.e., behind the RF coil) would also address this issue. Finally, we adopted a planar design of crusher coil to simplify manufacture. However, a curved design conforming to the subject's chest may further improve the ability to suppress skeletal muscle beyond that reported here.

Note also that the spoiling efficiency of the crusher coil was relative to the limited signal suppression provided by the current SAR‐limited BISTRO train pulse. This BISTRO train pulse was optimized for ^31^P‐MRS studies at 7T 8. However, it is possible that a more sophisticated RF pulse design could yield more robust suppression even with the very limited SAR and peak B_1_^+^ available for cardiac ^31^P‐MRS at 7T. Nevertheless, for many protocols, the advantages of zero‐SAR signal suppression with the crusher coil would still be enough to make the crusher coil the signal suppression method of choice.

Before we conclude, it is important to realize that the SAR reduction afforded by the crusher coil dramatically increases the flexibility of pulse sequence design for cardiac ^31^P‐MRS at 7T. For example, it will enable us to use all the allowed SAR in, e.g., adiabatic excitation pulses (or optimal control pulses) for absolute quantitation, or for ^1^H‐^31^P NOE enhancement or for frequency‐selective saturation pulses (or pulse trains) to probe creatine‐kinase kinetics.

## CONCLUSIONS

We conclude that a crusher coil is a simple‐to‐use, reliable, and effective alternative to BISTRO saturation for suppressing skeletal muscle during cardiac ^31^P‐MRS scans at 7T. The flexibility offered by using the crusher coil allows us to use sequence modules for future clinical studies at 7T that would otherwise be SAR‐prohibitive, without having to compromise on skeletal muscle suppression.

## Supporting information


**Figure S1**. Circuit diagram of the setup driving the current through the crusher coil. The entire setup was present in three main areas: the console room (dashed blue square), the scanner room (dashed red square) and within the magnet bore (dashed yellow square).
**Figure S2**. I_spoil_ time course profile for different driving PSU voltages. I_spoil_(t) was measured across a 0.1 Ω resistor. T_spoil_ was fixed at 100 μs.
**Figure S3**. Photograph of the phantom used for transmit RF field test calibration.
**Figure S4**. Apparatus used for peak integral comparison (left) and the overlap of the CSI grid on CINE FLASH sagittal (center) and transverse (right) images. The SNR was averaged over the yellow square (90 × 105 mm^2^).
