# Supplementary material for: Suppression of skeletal muscle signal using a crusher coil: A human cardiac 31p‐MR spectroscopy study at 7 tesla
Source: Magn Reson Med. 2015 Apr 28;75(3):962–72. doi: 10.1002/mrm.25755 (PMC4762536; doi:10.1002/mrm.25755)
Supplement: Supplementary file 1 — Figure S1. Circuit diagram of the setup driving the current through the crusher coil. The entire setup was present in three main areas: the console room (dashed blue square), the scanner room (dashed red square) and within the magnet bore (dashed yellow square). Figure S2. Ispoil time course profile for different driving PSU voltages. Ispoil(t) was measured across a 0.1 Ω resistor. Tspoil was fixed at 100 μs. Figure S3. Photograph of the phantom used for transmit RF field test calibration. Figure S4. Apparatus used for peak integral comparison (left) and the overlap of the CSI grid on CINE FLASH sagittal (center) and transverse (right) images. The SNR was averaged over the yellow square (90 × 105 mm2). [file MRM-75-962-s001.docx]

# Supporting information for Suppression of skeletal muscle signal using a crusher coil: a human cardiac ^31^P-MR spectroscopy study at 7 tesla

**Crusher coil control circuit**

A reservoir capacitor was used to drive the high I_spoil_ during a short T_spoil_ in the crusher coil. The entire setup is shown is Figure SI1. The capacitor (C = 2.5 mF) was placed in the magnet room, close to the filter panel, and was continuously charged via a power supply unit (PSU_1_, Iso-Tech IPS23002A) situated in the console room. Resistor R_2_ was used as a safety measure to allow the capacitor to discharge after use. The scanner’s fibre-optic trigger output was converted to TTL, fed to a microcontroller (Arduino Uno, AT mega 328 2009, constant power supply PSU_2_ = 10 V) and used to trigger I_spoil_ through the crusher coil. During T_spoil_, the capacitor discharged through the crusher coil. The current provided to the crusher coil was measured using an oscilloscope, plugged across a resistor (R_3_ = 0.1 Ω). To avoid any back-EMF produced in the metal–oxide–semiconductor field-effect transistor (MOSFET) as the current suddenly stops, a diode D_2_ and resistor R_4_ were used to remove the voltage from the inductive load (i.e. the crusher coil). A low-pass filter L (cut-off frequency 5 MHz) was placed before the crusher coil to minimize coupling between the crusher coil and the RF coil it is used with. The buffer circuit (two successive NAND gates here) reduced the current drain requirements on the Arduino’s output pins to be within their specified limits.

For safety, all circuit components were enclosed in boxes and connections were made using BNC connectors with grounded outer braiding. A foam sleeve was used to protect the volunteer and the user any possible common-mode currents on the outer sheath of the coaxial cable.

The wires between the console room and the magnet room were patched through low-pass filters (BLP-1.9+, MiniCircuits, New-York, USA) in the penetration panel to prevent RF pickup on the lines.


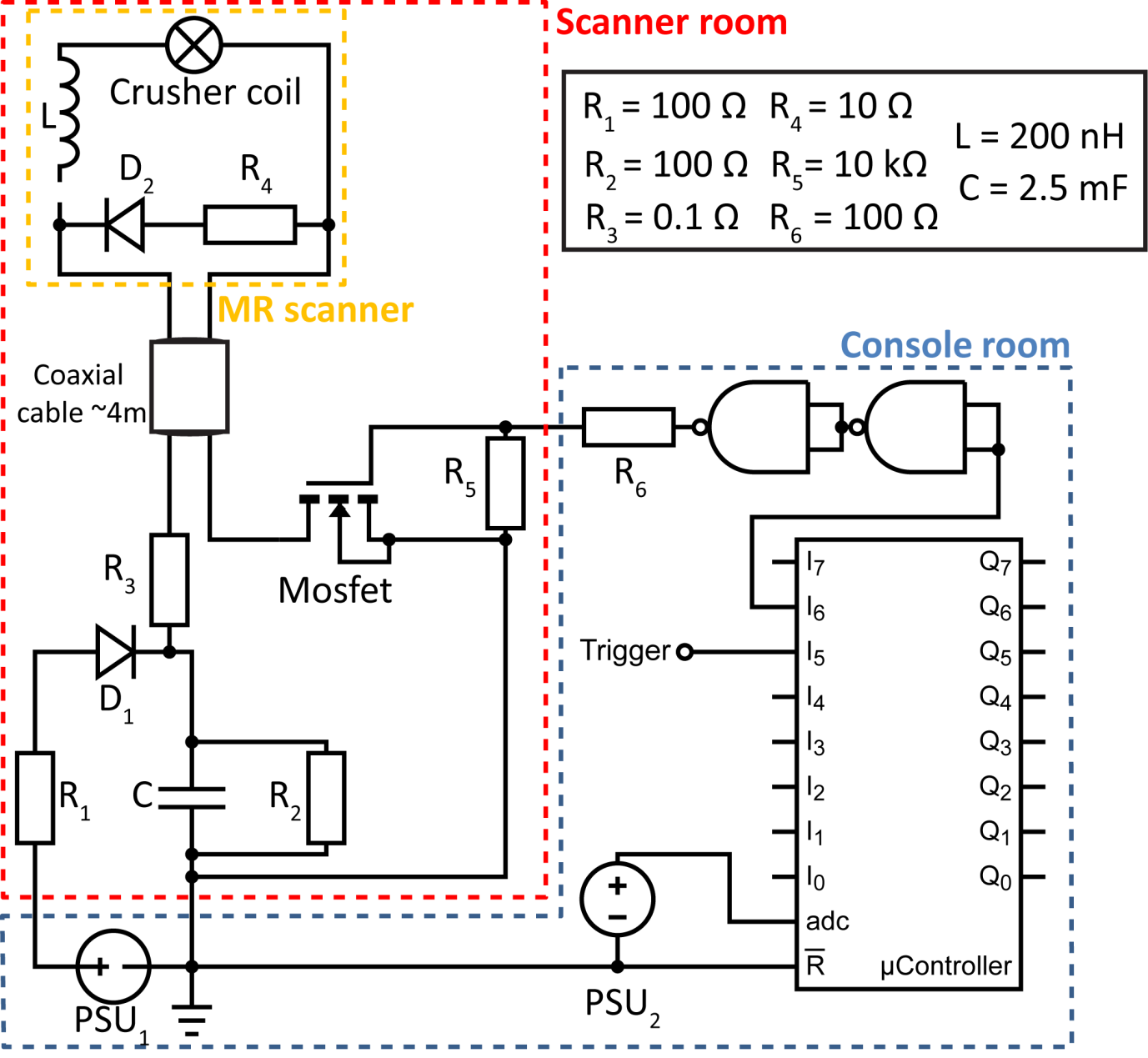


***Figure SI1:*** *Circuit diagram of the setup driving the current through the crusher coil. The entire setup was present in three main areas: the console room (dashed blue square), the scanner room (dashed red square) and within the magnet bore (dashed yellow square).*

**I_spoil_ time course**

The current through the crusher coil I_spoil_(t) was checked using an oscilloscope (Picoscope 2204A) to measure the voltage drop across an 0.1 Ω resistor temporarily connected in series with the crusher coil. I_spoil_ was measured for PSU voltages from 5 to 20V. The rise and fall times of the current profiles were < 50 μs in all cases. Pulse profiles were consistent from shot-to-shot (Fig. SI2). The pulse timing jitter was < 10 μs in all instances (this was mainly due to the Arduino **
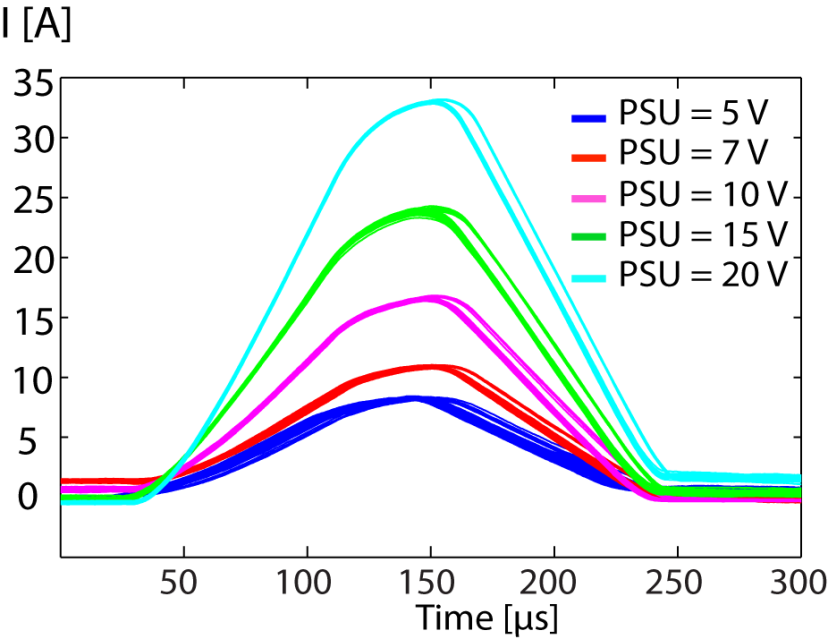
**clock)

***Figure SI2:*** *I_spoil_ time course profile for different driving PSU voltages. I_spoil_(t) was measured across a 0.1 Ω resistor. T_spoil_ was fixed at 100 μs.*

**RF safety test**

To confirm RF safety before use with volunteers, we followed an established protocol (1). The crusher coil was placed between the 10cm ^31^P RF coil and 2kg of beef for loading, the setup was left for 15min for temperature equilibration, a FID pulse sequence (TR = 1 sec, maximum RF voltage (100% nominal SAR), RF pulse duration = 1.25 ms) was run for ~30min, and the temperature rise produced by this sequence was monitored to ensure compliance with IEC guidelines during human scans (SAR < 10 W/kg, “Normal controlled mode” for a local transmit coil in the trunk) (2). The experiment was performed with and without the crusher coil in place, and was repeated twice. Two fibre optic temperature probes (Neoptix Reflex Inc., Canada) were inserted into the sample. One probe was placed under the position of the capacitors (i.e. at the location of maximum predicted SAR) and one probe was placed in the centre of the ^31^P RF coil. The temperature probes were placed ~5 mm beneath the surface of the meat. The temperature change over time was recorded from both probes with an accuracy of 0.1°C at a time resolution of 1 sample per second.

During these tests, the crusher coil was operated with I_spoil_×T_spoil_ = 11 A·ms, which is ~10× more saturation than used *in vivo*:

RF heating with RF coil alone:$max(\frac{d\Delta T}{dt})$ = 0.038^o^C/min

RF heating with RF coil and crusher coil:$max(\frac{d\Delta T}{dt})$ = 0.051^o^C/min

Considering a meat heat capacity of 4200 J/kg, the maximum SAR was 2.7 W/kg (RF coil) and 3.6 W/kg (RF coil + crusher coil). These values were far below the 10 W/kg, which corresponds to “Normal controlled mode” for a local transmit coil in the trunk (2). According to these calculations, the use of the crusher coil (in addition to the RF coil) does not lead to a substantial increase of temperature change and was therefore safe to use.

Additionally, a sheet printed with liquid crystal ink that changes colour with temperature (Edmund Optics, Inc. #72-375 sheets) was placed between the meat and the crusher coil. No visual differences were distinguishable between using the RF coil alone, or using the RF coil and crusher coil together. This suggests that the crusher coil did not introduce new heating hot-spots.

As a final heating test, we did a continuous 40 A discharge (i.e. the maximum supported by the power supply) through the crusher coil for 10 min (on the bench, without the TX/RX coil) and we did not detect any warming of the wires.

This data was reviewed by the local coil safety committee and the crusher coil was therefore deemed safe for human use at 7T.

**Transmit RF field test**


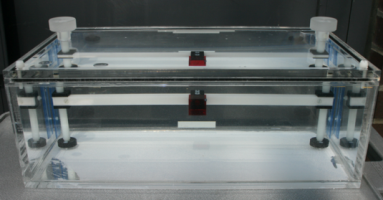
A transmit RF field (B_1_^+^) mapping experiment was performed in the scanner as follows: a pulse-acquire MR pulse sequence (TR = 10 sec, 4 preparation scans, NT = 3, RF duration = 4 ms) was run with various RF excitation voltages ranging from 50 V to 270 V. A phantom filled with 85% H_3_PO_4_ was placed 60 mm away from the RF coil (red cube in Fig. SI3). A matching distance between the ^31^P RF coil and the phantom loading was ensured when changing the experimental setup (i.e. sliding the crusher coil in between the RF coil and the phantom). The RF coil tuning and matching was checked and found to be comparable with and without the crusher coil. Each test was run twice to gauge reproducibility. Results are presented in the manuscript. The experiment was also run with the RF coil alone (i.e. no padding was used to replace the crusher coil).

***Figure SI3:*** *Photograph of the phantom used for transmit RF field test calibration.*

**SNR variation test**

To investigate the SNR variation without and with the crusher coil in place, a 3D-CSI experiment was performed with a single slice phantom (120×180×35 mm^3^, on the sagittal plane). The sequence parameters were: TR = 1000 ms, TE = 2.3 ms, matrix size = 240×240×100 mm^3^, resolution = 16×16×4, TA = 4 min 30. The setup is shown in Fig. SI4. When the crusher coil was not physically present, a foam pad was placed between the RF coil and the loading to keep the RF coil in the same position. The CSI grid was placed in the sagittal plane. ^31^P-MRSI was first performed without the crusher coil and then the crusher coil was inserted. The SNR was calculated for each setup, for each voxel, and averaged over the yellow square area drawn in Figure SI4 (90×105mm^2^), which corresponds to the area below the ^31^P RF coil. Results are presented in the manuscript.


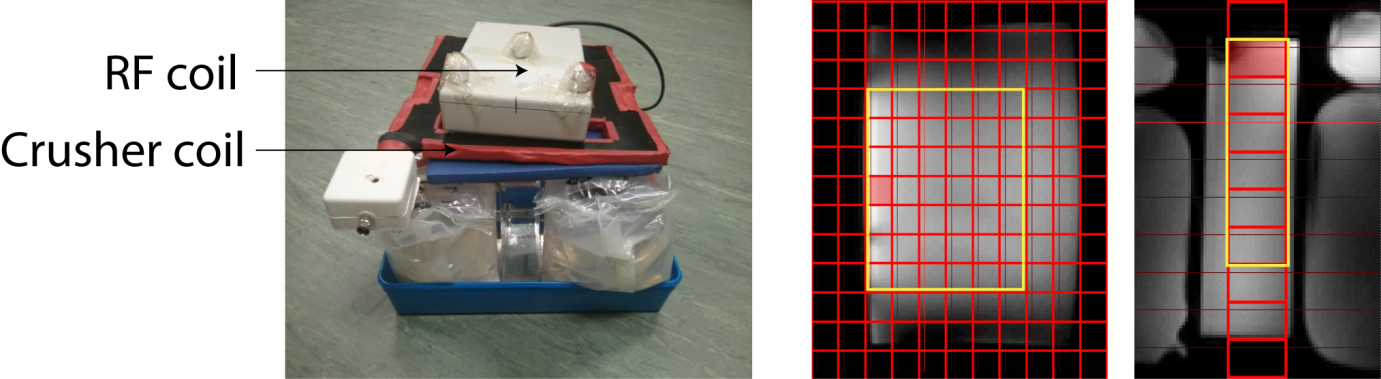


***Figure SI4:*** *Apparatus used for peak integral comparison (left) and the overlap of the CSI grid on CINE FLASH sagittal (centre) and transverse (right) images. The SNR was averaged over the yellow square (90×105mm^2^).*

## References:

1. El-Sharkawy AM, Schar M, Ouwerkerk R, Weiss RG, Bottomley PA. Quantitative cardiac 31P spectroscopy at 3 Tesla using adiabatic pulses. Magn Reson Med 2009;61(4):785-795.

2. British Standard. Medical electrical equipment: International Standards IEC 60601. 2010;Part 2-33.
